# A closed-loop strategy for composting: using biochar and fulvic acid derived from manure or straw to mitigate ammonia emission and enhance humification

**DOI:** 10.1039/d5ra08201c

**Published:** 2026-02-03

**Authors:** Chihao Yang, Jing Wu, Wenping Xie, Jingsong Yang, Yanfang Feng, Xiangping Wang, Xin Zhang, Xuan Yu, Rongjiang Yao

**Affiliations:** a State Key Laboratory of Soil and Sustainable Agriculture, Institute of Soil Science, Chinese Academy of Sciences Nanjing 210008 China wpxie@issas.ac.cn; b Key Laboratory of Agro-Environment in Downstream of Yangtze Plain, Institute of Agricultural Resources and Environment, Jiangsu Academy of Agricultural Sciences Nanjing 210014 China; c College of Energy and Environment, Anhui University of Technology Maanshan 243002 China

## Abstract

Biochar (BC) and biostimulants, such as fulvic acid (FA), have proven to have potential in composting. They have been shown to reduce nitrogen loss and enhance the quality of compost, benefitting the composting of manure and straw. However, the synergistic effects of BC and FA on composting remain largely unelucidated. The present study evaluated the individual and synergistic effects of BC and FA on the composting of chicken manure mixed with straw. Results demonstrated that BC + FA significantly reduced ammonia (NH_3_) volatilization by 48.11% through the composting process. In addition, BC + FA increased the accumulated temperature and pH and reduced the electrical conductivity of composts. Regarding the final products, the BC + FA treatment increased the contents of total nitrogen and dissolved organic carbon and improved the germination index (GI) by 50%. Concurrently, FA and BC + FA increased the content of humic substances by 12.6% and 12.8%, respectively. The ratio of humic acid to FA increased from 8.2% to 44.9% following the BC + FA treatment compared to that in the control. Furthermore, BC enhanced the fluorescence and humification indices of the composts. Besides, it was revealed that the functional groups present on the surfaces of BC and FA + BC were associated with intermolecular polymerization and aromatization. The Mantel test confirmed that BC + FA effectively reduced the NH_3_ emissions of this process and enhanced the quality and GI, probably through stimulating the directional transformation of organic matter. This study systematically evaluated the effect of BC and FA in a composting trial and offered a promising and comprehensive strategy for the effective resource utilization of manure and straw.

## Abstract 2

The combined application of biochar (BC) and fulvic acid (FA) has shown potential for mitigating ammonia emission and enhancing the process of composting.

## Graphical abstract

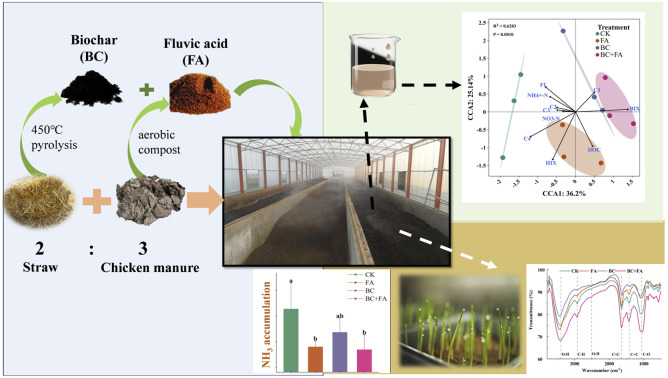

## 1. Introduction

The global demand for low-cholesterol meat has increased significantly, resulting in a substantial expansion of the poultry industry.^1^ According to the National Bureau of Statistics of China, approximately 3.8 billion tons of livestock manure are produced annually in China, with poultry manure comprising nearly 40% of this amount.^2,3^ Poultry manure is widely used in agriculture because it is characterized by an abundance of organic matter and essential nutrients such as nitrogen (N) and phosphorus (P). Indeed, it was reported that its use can potentially reduce the reliance on inorganic fertilizers by approximately 30%.^4^ However, the direct application of untreated poultry manure can raise various environmental concerns, including air and water pollution, the release of phytotoxic compounds, and the spread of human pathogens.^1^

Composting represents an effective and economical solution for treating poultry manure, which can be used as an organic fertilizer.^5^ During the process of composting, the decomposition of unstable organic matter by microorganisms results in the transformation of this material into mature, stable composts that can be used in farming.^6^ The key quality indicators for composts include maturity level, nutrient content, organic matter percentage, pH, and germination index.^7^ However, limitations of composting include the release of greenhouse gases and other harmful gases.^8^ It can also propagate some pollutants and pathogens. In addition, nutrient deficiencies, particularly nitrogen loss, influences the quality of composts. This is mostly because of NH_3_ volatilization during this process.^2^ Consequently, optimizing the treatment of poultry manure in the composting process is imperative to enhance its suitability and effectiveness for agricultural applications.^9^

In order to enhance the efficiency of composting and reduce its environmental impact, the addition of physical, chemical, and bioactive substances has been shown to be effective, which optimizes the process.^10^ Among these additives, biochar (BC) has gained attention owing to its unique structure and remarkable physicochemical properties.^11^ BC is a solid product formed by the pyrolysis of organic matter under conditions of low oxygen content at temperatures below 900 °C. The substance under scrutiny is predominantly composed of aromatic molecules, and it possesses a high specific surface area, and contains oxygen-functional groups on its surface.^12^ These characteristics render BC an effective additive in composting, with the capacity to regulate pH, enhance nutrient retention, and reduce gas emission.^13,14^ Furthermore, BC has been reported to reduce the bioavailability of heavy metals, degrade organic pollutants, and remove antibiotic resistance genes through a range of mechanisms including adsorption, complexation, co-precipitation, and microbial activation.^1,11,15^

In recent years, there has been increasing research focus on the use of biostimulants such as fulvic acid (FA) in the context of composting.^16^ FA is characterized by a high content of soluble organic acids including carboxyl and phenolic groups. This compound shares similarities with biochar in terms of its porous structure, but it possesses a more complex molecular composition and a greater abundance of functional groups.^17,18^ These features integrated the complementary benefits of physical, chemical, and biological additives.^19,20^ Although microorganisms activated during composting can synthesize FA, this typically occurs in the mid-to late-stages, limiting its availability during the critical early phase, and thereby, constraining the overall composting efficiency.^16^ It was demonstrated that the addition of small amounts of FA reduced gas emissions, minimized nitrogen loss, and increased the germination index (GI) in composting.^10^ Other studies have shown that FA or FA-containing substances significantly altered the microbial community structure and accelerated the composting process.^10,21^

Both BC and FA are typically derived from manure or straw. As distinct approaches to managing these organic wastes, we hypothesized that the application of BC and FA in composting could represent a synergistic strategy for the integrated utilization of organic wastes. This study aimed to assess the effectiveness of BC and FA in accelerating the composting process and improving the compost quality. Furthermore, we investigated their combined effects and identified the key factors of BC and FA influencing composting outcomes, aiming to provide valuable and practical insights for optimizing chicken manure composting.

## 2. Materials and methods

### 2.1. Experimental materials and design

Fresh chicken manure was obtained from Musen Fertilizer Co., Ltd, and wheat straw was obtained from Huifeng Straw Agricultural Products Deep Processing Co., Ltd. Biochar (BC), produced from wheat straw *via* pyrolysis at 450 °C, was purchased from Nanjing Qinfeng Straw Technology Co., Ltd. Fulvic acid (FA) was supplied by Beijing Bowei Shennong Technology Co., Ltd.

Chicken manure and wheat straw were mixed at a fresh weight ratio of 1.5 : 1 to achieve an initial C/N ratio of approximately 25, following established co-composting methods.^22,23^ Prior to composting, proper deionized water was added to adjust the initial moisture content to 60%. Composting was conducted in custom-made insulated bins (640 × 490 × 70 mm^3^) with a polypropylene exterior and an expanded polystyrene foam core.

Based on the previous studies,^10,14^ four treatments were established: CK (control without additive), FA (mixed manure with 3% (w/w) FA), BC (mixed manure with 15% (w/w) BC), and BC + FA (mixed manure with 3% (w/w) FA and 15% (w/w) BC). During the composting process, the moisture content in all treatments was maintained at approximately 60% by weight. The compost was turned thoroughly on days 1, 4, 7, 10, 13, 17, 21, and 28, with the entire period set at 28 days.

### 2.2. Sample collection and analysis

Compost samples were collected on days 1, 4, 7, 10, 13, 17, 21, and 28. After thorough turning, the representative samples were immediately collected, sealed, and stored at −20 °C for subsequent analyses.

#### 2.2.1. Temperature record

Throughout the composting period, temperature fluctuations at the upper, middle, and lower sections of the compost were monitored using a temperature recorder (RC-4). The average temperature was calculated. The effective accumulated temperature (EAT) was calculated using the following formula (eqn (1)):1
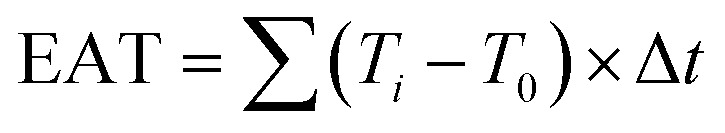


*T*_*i*_ represents the compost temperature at time *i*, *T*_0_ is the initial temperature at which microbial activity begins to proliferate in the compost (biological zero), and Δ*t* is the duration for which *T*_*i*_ persists. For this study, a biological zero of 15 °C was used to represent the temperature threshold at which composting reactions and microbial activity begin to accelerate.

#### 2.2.2. NH_3_ volatilization during composting

Ammonia (NH_3_) gas was collected daily for the first 24 days of composting, and then every two days thereafter until day 28. NH_3_ was captured at the gas outlet of the fermentation tank using a 2% boric acid solution. The NH_3_ concentration was then titrated with 0.01 M dilute sulfuric acid, and the cumulative NH_3_ volatilization flux was calculated. This method follows the approach described in a previous study.^24^

The NH_3_ volatilization flux was calculated using the following formula (eqn (2)):2
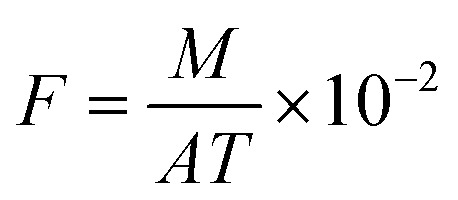
where *F* represents the NH_3_ volatilization flux (kg hm^−2^ day^−1^); *M* indicates the average amount of NH_3_ measured per device during each capture (mg); *A* denotes the cross-sectional area of the capture device (m^2^); and *T* refers to the duration of each continuous capture (days).

Additionally, the cumulative NH_3_ volatilization flux (CF) can be determined using the following equation (eqn (3)):3
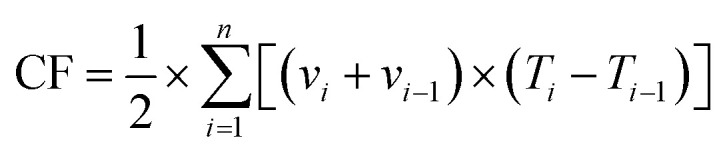
where CF represents the cumulative NH_3_ volatilization (kg hm^−2^), *n* denotes the total number of sampling events, *v*_*i*_ is the NH_3_ volatilization rate of the *i*-th sample, and *T* indicates the duration in days for the *i*-th sampling event.

#### 2.2.3. Quality analysis of composts

Approximately 2 g of fresh compost sample was mixed with deionized water at a ratio of 1 : 10 (w/v). The mixture was shaken for 30 minutes, settled for 10 minutes, and then centrifuged at 3200 rpm for 30 minutes. The resulting supernatant was collected for analysis.^2^ The pH and electrical conductivity (EC) of the filtrate were measured using a multi-parameter analyzer.

Total nitrogen (TC) and total nitrogen (TN) in the dried compost samples were measured using an elemental analyzer. Total phosphorus (TP) was determined using inductively coupled plasma optical emission spectroscopy (ICP-OES, Optima™ 8000, PerkinElmer). For inorganic nitrogen analysis, the fresh compost sample was extracted with a 2 mol L^−1^ KCl solution (1 : 10, w/v) by shaking for 60 minutes, followed by standing for 10 minutes and filtration. Ammonium nitrogen (NH_4_^+^-N) and nitrate nitrogen (NO_3_^−^-N) concentrations in the extract were quantified using a continuous-flow analyzer (SFA, San++, Skalar).^25^ The changes in the functional groups of compost samples were analyzed using Fourier transform infrared (FTIR) spectroscopy (Nicolet 6700, Thermo Scientific, USA).

The germination index (GI) of each compost sample was determined^26^ using the following formula (eqn (4)):4
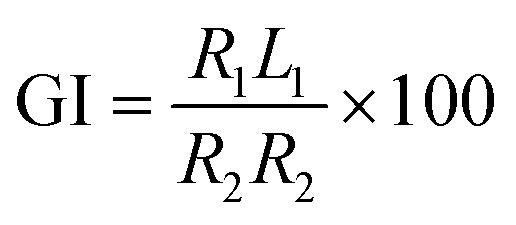
where *R*_1_ is the seed germination rate (%) in the compost leachate, *L*_1_ is the root length (mm) of seeds in the compost leachate, *R*_2_ is the seed germination rate (%) in distilled water, and *L*_2_ is the root length (mm) of seeds in distilled water.

#### 2.2.4. Analysis of organic matter

For the composts, 1.0000 g of dried and ground sample was accurately weighed into a pre-tared crucible. The crucible was placed in a muffle furnace and heated at 450 °C for 8 hours until a constant weight was achieved.^26^ After cooling to room temperature, the residue was weighed to calculate the organic matter (OM) content. The OM content was calculated using the following formula (eqn (5)):5
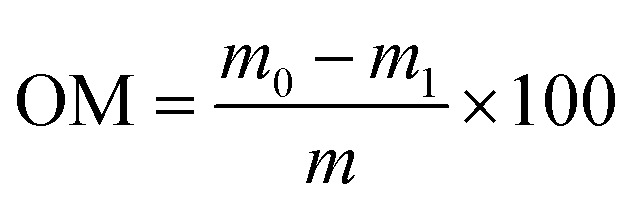
where *m*_0_ is the weight of the dry sample before ignition (g), *m*_1_ is the weight of the sample after ignition (g), and *m* is the weight of the dry sample (g).

The total organic carbon (TOC) content of humic substances (HSs), humic acid (HA), and FA was measured using a total organic carbon analyzer (TOC-L CPH, Shimadzu, Japan). The detailed sample pretreatment and measurement procedures are presented in Text S1.^27^

To extract dissolved organic matter (DOM), 5 g of the final compost product was mixed with deionized water at a ratio of 1 : 10 (w/v). The mixture was shaken for 24 hours and then centrifuged at 6000 rpm for 15 minutes. The supernatant was filtered through a 0.45 µm filter membrane, and the filtrate was collected as the compost DOM sample. The dissolved organic carbon (DOC) content of the extract was determined using a total organic carbon analyzer (TOC-L CPH, Shimadzu, Japan). The excitation-emission matrix (EEM) fluorescence spectra were recorded using a FluoroLog-3 spectrometer. The spectra were modeled by parallel factor analysis (PARAFAC) to resolve independent fluorescence components, which allowed the tracking of DOM source and compositional changes. Four fluorescence components were identified: C1 (*E*_*x*_/*E*_*m*_ = 350 nm/440 nm), C2 (*E*_*x*_/*E*_*m*_ = 395 nm/494 nm), C3 (*E*_*x*_/*E*_*m*_ = 295 nm/418 nm), and C4 (*E*_*x*_/*E*_*m*_ = 330 nm/396 nm). All spectra were Raman-normalized according to the previous research.^28^ The fluorescence index (FI), biological index (BIX), and humification index (HIX) were calculated from the EEM data to further characterize the DOM.

### 2.3. Statistical analysis

Data processing was performed using Microsoft Excel (2019), while statistical analysis was conducted with SPSS 16.0. Significant differences were determined using Duncan's multiple range test through one-way analysis of variance (ANOVA) (*P* < 0.05). Images were created using Origin (2019b); Mantel Test and Structural Equation Modeling (SEM) were generated using R (4.2.1) and Adobe illustrator 2024; 3D-EEM images were generated using R (4.2.1).

## 3. Results

### 3.1. Changes in physicochemical parameters during the composting process

**Fig. 1:**
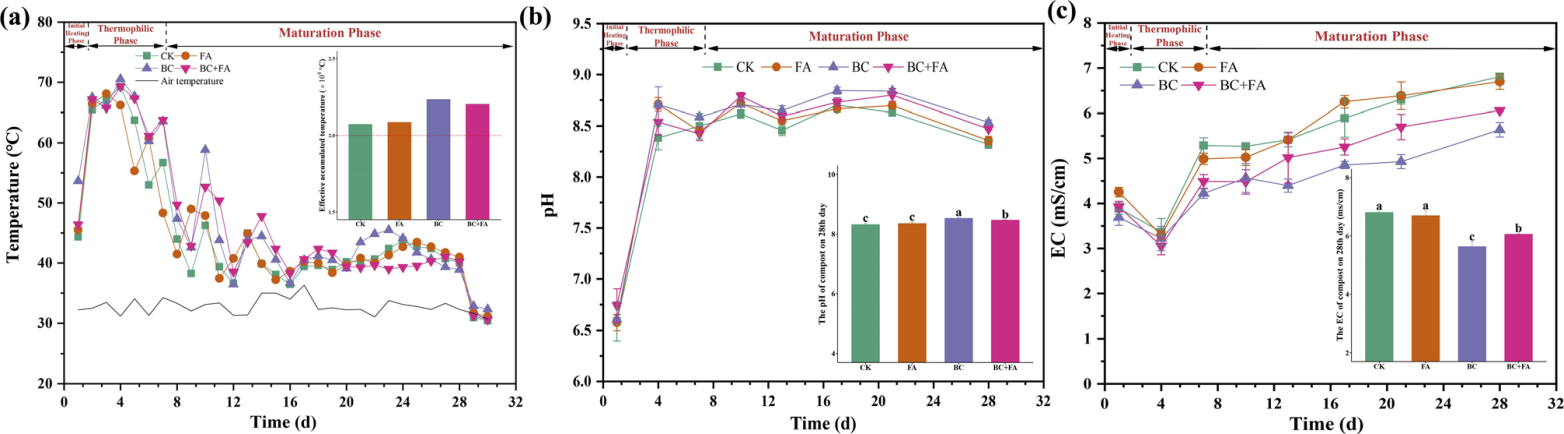
Dynamics of temperature (a), pH (b), and EC (c) during composting. Data are presented as mean ± SD (*n* = 3), and the different lowercase letters above the columns indicate significant differences at *P* < 0.05.

As illustrated in Fig. 1a, the ambient temperature during composting ranged from 30.7 °C to 36.3 °C. In all treatments, the compost temperature progressed through three typical phases-initial heating, thermophilic, and maturation. Peak temperatures (68.1 °C and 70.5 °C) were reached within the first week, after which temperatures gradually declined and stabilized near ambient levels. The CK, BC, and BC + FA treatments attained their highest temperatures on day 4, while the FA treatment peaked earlier, on day 3, at a lower maximum than the other treatments. The effective accumulated temperature was higher in all treatments compared to CK, with the BC treatment recording the greatest value (21 985.5 °C).

As shown in Fig. 1b, the initial compost pH ranged from 6.57 to 6.74 and then increased rapidly to between 8.38 and 8.71 within the first four days. From day 7 onward, the pH remained stable in the range of 8.42 and 8.84. During the thermophilic phase, the pH of the FA and BC treatments was significantly higher than that of CK (*P* < 0.05). Toward the end of this phase, all treatments except CK showed a decline in pH to varying degrees. By the maturation phase and throughout the remainder of composting, pH followed the order of BC > BC + FA > FA > CK, with the BC and BC + FA being significantly higher than CK.

As illustrated in Fig. 1c, the EC of all treatments increased from an initial range of 3.69 to 4.25 mS cm^−1^ to a final range of 5.64 to 6.81 mS cm^−1^ by the end of composting. On day 4, EC dropped to a minimum of 3.05 to 3.37 mS cm^−1^, after which it increased to 4.22–5.29 mS cm^−1^ by the end of the thermophilic phase. During maturation, EC continued to increase gradually until composting was complete. No significant differences in EC were observed among the treatments at minimum point. However, from the late thermophilic phase onward, the EC of the CK and FA treatments was significantly higher than that of the BC and BC + FA treatments (*P* < 0.05).

### 3.2. Changes in NH_4_^+^-N, NO_3_^−^-N and NH_3_ volatilization during the composting process

**Fig. 2:**
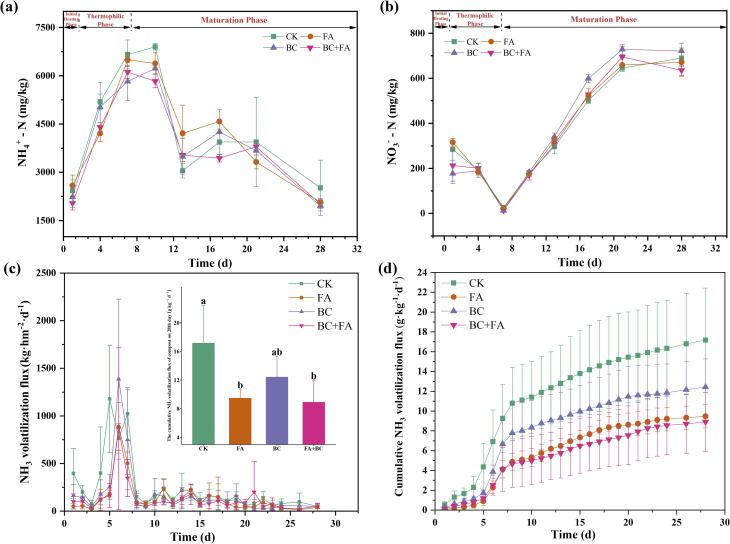
Dynamics of NH_4_^+^-N level (a) and NO_3_^−^-N level (b). NH_3_ volatilization flux (c) and cumulative NH_3_ volatilization flux (d) during composting. Data are presented as mean ± SD (*n* = 3), and different lowercase letters above the columns indicate statistically significant differences at *P* < 0.05.

As shown in Fig. 2a, the NH_4_^+^-N levels in all treatments increased significantly from day 1 until the end of the thermophilic phase (day 7). After reaching their peak, the NH_4_^+^-N concentrations dropped rapidly between days 10 and 13, fluctuated until day 21, and then returned to their initial levels by the end of composting (day 28). The highest NH_4_^+^-N levels were observed on day 10 in the CK and BC treatments. However, in the FA and BC + FA treatments, the peak occurred earlier, on day 7. Throughout the thermophilic and early maturation phase, the CK treatment maintained the highest NH_4_^+^-N levels among all treatments. By the end of composting, the FA, BC, and BC + FA treatments reduced NH_4_^+^-N contents by 17.57%, 22.16%, and 20.12%, respectively, compared to CK.

In contrast, the NO_3_^−^-N levels (Fig. 2b) reached their lowest point across all treatments at the end of the thermophilic phase (day 7) and then increased steadily until day 21. During the maturation phase, the BC treatment consistently exhibited higher NO_3_^−^-N levels than the other treatments. By the end of composting, BC treatment increased the NO_3_^−^-N levels by 4.64%, and the FA and BC + FA treatments reduced NO_3_^−^-N contents by 2.70% and 7.84%, compared to CK.

The trend in NH_3_ volatilization flux was generally consistent across all treatments (Fig. 2c). During the initial heating and thermophilic phases, the lowest daily flux (24.08–74.90 kg hm^−2^ day^−1^) for all treatments occurred on day 3, while the peak values (765.10–1385.73 kg hm^−2^ day^−1^) were observed on day 6, except in the CK treatment. Before the maturation phase, the NH_3_ flux in CK was generally higher than that in the other treatments, with exception of day 6. Following the onset of maturation, the NH_3_ flux in all treatments decreased and remained low until the end of composting. Cumulative NH_3_ volatilization was reduced by 44.85%, 27.64%, and 48.11% in the FA, BC, and BC + FA treatments, respectively, compared to CK (Fig. 2c). Accordingly, cumulative NH_3_ emissions followed the order of BC + FA < FA < BC < CK (Fig. 2d).

### 3.3. The Mantel test of NH_3_ volatilization and pH, EC, NH_4_^+^-N, NO_3_^−^-N in different compost stages

**Fig. 3:**
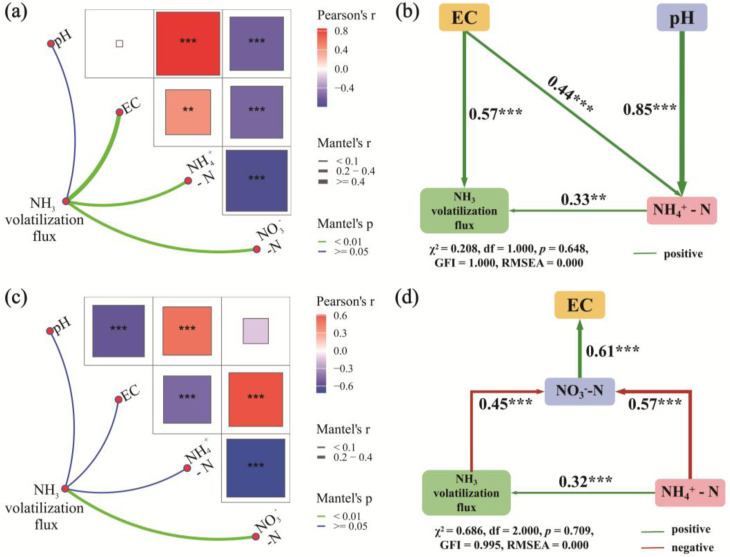
Mantel test results and SEM analysis of the relationships between NH_3_ volatilization and various factors (pH, EC, and NH_4_^+^-N) in the thermophilic phase (a and b) and maturation phase (c and d).

During the initial heating and the thermophilic phase, the Mantel test results (Fig. 3a) indicated that NH_3_ volatilization flux was primarily correlated with EC (*r* = 0.50, *P* < 0.001), NH_4_^+^-N levels (*r* = 0.29, *P* < 0.001), and NO_3_^−^-N levels (*r* = 0.34, *P* < 0.001) but not with pH. The NH_4_^+^-N levels were positively correlated with the pH (*r* = 0.85, *P* < 0.001) and EC (*r* = 0.43, *P* < 0.01), and negatively associated with NO_3_^−^-N levels (*r* = −0.79, *P* < 0.001). In turn, the NO_3_^−^-N levels were negatively correlated with the pH (*r* = −0.61, *P* < 0.001) and EC (*r* = −0.58, *P* < 0.001). SEM analysis (Fig. 3b) further indicated that NH_4_^+^-N levels explained 33% and EC explained 57% of the variation in NH_3_ volatilization (*P* < 0.01).

During the maturation phase, NH_3_ volatilization flux was primarily driven by NO_3_^−^-N levels (*r* = 0.33, *P* < 0.001), with no significant correlation observed with pH, EC, or NH_4_^+^-N levels (Fig. 3c). The NH_4_^+^-N levels remained positively correlated with the pH (*r* = 0.50, *P* < 0.001) but showed negative correlations with the NO_3_^−^-N levels (*r* = −0.72, *P* < 0.001) and EC (*r* = −0.48, *P* < 0.001). Conversely, EC was negatively correlated with the pH (*r* = −0.56, *P* < 0.001) and positively correlated with the NO_3_^−^-N levels (*r* = 0.61, *P* < 0.001). SEM analysis (Fig. 3d) further revealed that NH_4_^+^-N levels explained 32% of the variation in NH_3_ volatilization flux (*P* < 0.001), while the NO_3_^−^-N levels showed a positive contribution to EC (*r* = 0.32, *P* < 0.01).

### 3.4. Enhancement of compost quality by BC or/and FA

**Fig. 4:**
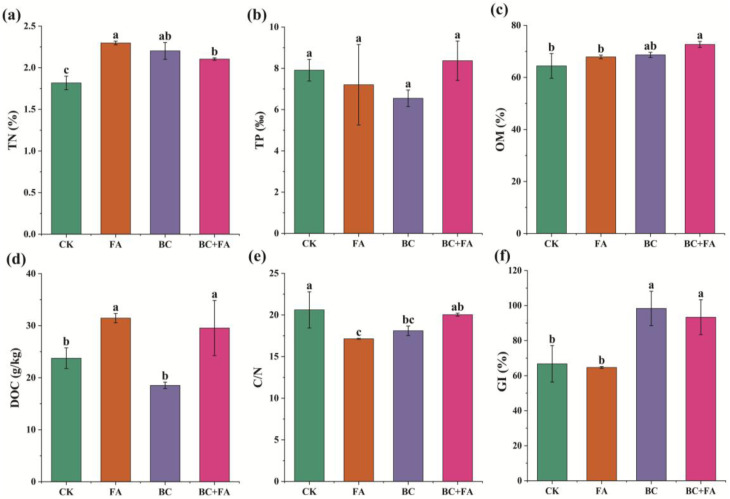
TN (a), TP (b), OM (c), DOC (d), C/N ratio (e) and GI (f) of the compost. Data are presented as mean ± SD (*n* = 3), and the different lowercase letters above the columns indicate statistically significant differences at *P* < 0.05.

Following composting, the TN content in the FA, BC, and BC + FA treatments was significantly higher than that in CK, with the FA treatment exhibiting the highest TN content (2.30%; Fig. 4a). In contrast, no significant differences in the TP content were observed among the treatments (Fig. 4b). The OM content was higher in the BC + FA treatment compared to the FA and BC treatments, and it was significantly increased compared to CK (Fig. 4c). Furthermore, the FA treatment exhibited higher levels of DOC than those of the other treatments (Fig. 4d). The C/N ratio across all treatments ranged from 17.14 to 20.61, with the FA and BC treatments showing significantly lower values than those of CK (Fig. 4e). The GI for each compost treatment is illustrated in Fig. 4f. Both BC and BC + FA treatments exhibited significantly higher GI than CK and FA.

**Fig. 5:**
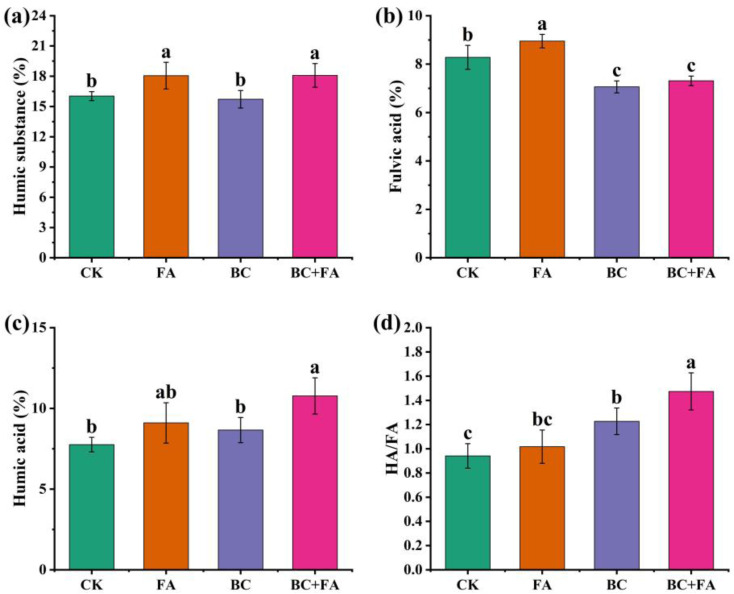
Contents of humic substances (HSs, a), fulvic acid (FA, b), and humic acid (HA, c) in the compost, and HA/FA ratio (d) of the compost. Data are presented as mean ± SD (*n* = 3), and the different lowercase letters above the columns indicate significant differences at *P* < 0.05.

The HS content in the final compost is shown in Fig. 5a. Both the FA and BC + FA treatments exhibited a significantly higher HS content than that of the CK and BC treatments. The fulvic acid content was significantly higher in the FA treatment than in the CK treatment. However, the fulvic acid content was significantly lower in the BC and BC + FA treatments than in the CK treatment (Fig. 5b). Among all treatments, the HA content was highest in the BC + FA treatment, showing a significant increase by 38.91% over CK and BC (Fig. 5c). Furthermore, the HA/FA ratio was significantly higher in the BC and BC + FA treatments than in the CK treatment (Fig. 5d).

### 3.5. Changes in the dissolved organic matter in the compost

**Fig. 6:**
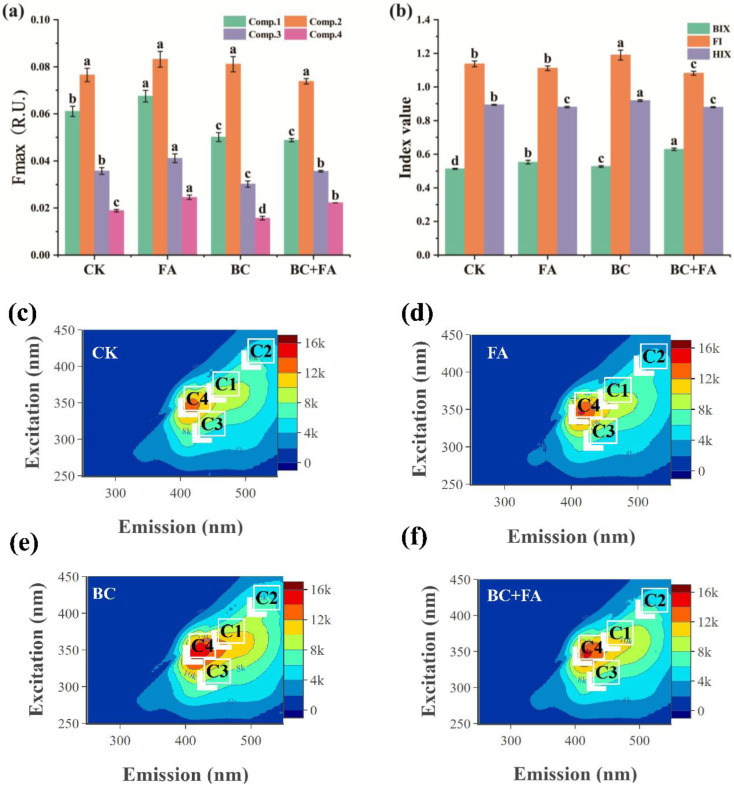
Maximum fluorescence intensities (*F*_max_) of the four components (a), fluorescence parameters of dissolved organic matter (DOM) (b), and EEM fluorescence spectra of DOC-fractions derived from different composts (c)–(f).

The fluorescence spectra of DOM in the compost were characterized using PARAFAC analysis. Fig. 6a illustrates the relative abundances of four components: C1, which corresponds to humic-like substances that probably originated from sewage;^29^ C2, which corresponds a rarely observed associated with dark incubation in lake environments;^30^ C3, which represents the terrestrial-derived humic substances (HSs);^31^ and C4, associated with marine-source HSs.^32^ BC and FA significantly altered the fluorescence intensities of DOM components. Compared with CK, the FA treatment increased the intensities of C1, C3, and C4 by 10.56%, 15.12%, and 30.21%, respectively. The BC treatment decreased the intensities by 17.89%, 15.51%, and 16.91%, respectively. Under the treatment of BC + FA, the C4 intensity was significantly increased, while the C3 intensity was significantly reduced. No significant change was observed in C2 across all treatments. The detailed EEM-FRI spectra reflected the composition of DOC in the compost, with the changes in C1, C2, C3 and C4 (Fig. 6c–f).

To further evaluate the impact of BC and FA on the compost quality, the fluorescence parameters BIX, FI, and HIX were calculated (Fig. 6b). Compared with CK, all addition treatments showed significantly higher BIX, with the BC + FA treatment exhibiting the highest BIX (0.6298)-significantly greater than those of the other treatments. The FI and HIX values were significantly elevated in the BC treatment compared to the CK treatment, whereas the BC + FA treatment showed the opposite pattern. Moreover, HIX in the FA treatment was significantly lower than that in the CK treatment.

### 3.6. Interaction between the indicators for solid phase and soluble aqueous phase in the compost

**Fig. 7:**
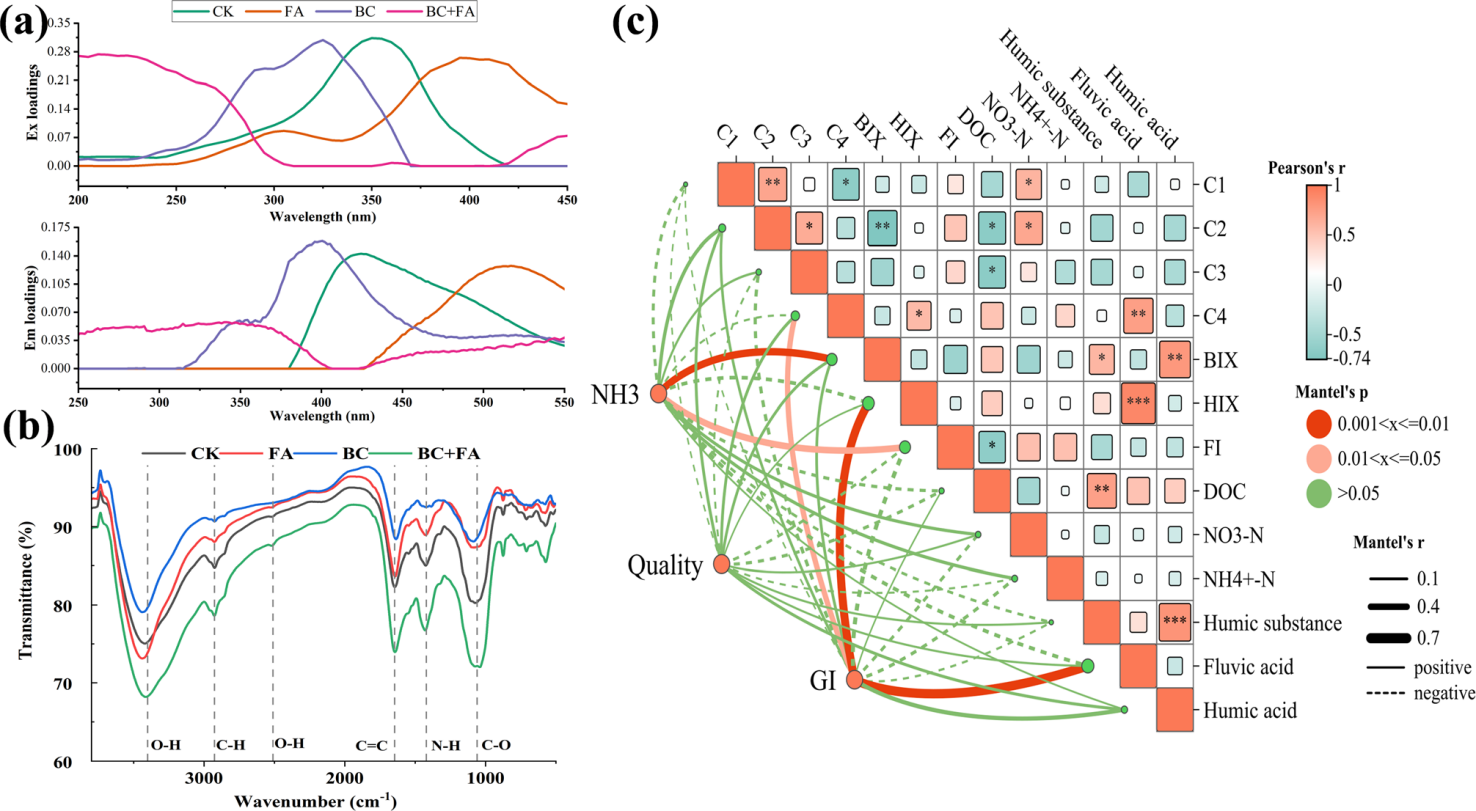
Loadings of DOM from compost-liquid (a), FIRT of compost-solid (b), and Mantel tests (c) showing the correlations of NH_3_ volatilization, GI and quality (OM, TN, TP, and TK) with dissolved organic particles (C1, C2, C3, C4, HIX, BIX, FI, NH_4_^+^-N, NO_3_^−^-N, humic substances, fulvic acid, and humic acid) in the compost.

Traditionally, FA has been used as an additive in composting and BC was considered as a carbon-based conditioner. The combined effect and underlying mechanism of FA and BC might involve multiple interactions. First, the excitation and emission loadings of DOM derived from different treatments are shown in Fig. 7a. Compared with CK, FA delayed the peak positions of the main loadings, while BC shifted them early. The combined BC + FA treatment exhibited a pattern similar to that of BC, but with earlier and broader peaks.

FIRT spectra was used to show the changes in functional groups of compost samples under different treatments (Fig. 7b). The characteristic peaks at 3400 and 2510 cm^−1^, corresponding to the O–H bond stretching in alcoholic hydroxyl and acidic hydrogen groups, were decreased in the BC treatment.^23,33,34^ The peak at 2925 cm^−1^, related to the –CH_3_ vibration, also weakened in the BC treatment.^33^ In contrast, the aromatic ring C=C stretching vibration near 1645 cm^−1^ intensified in the BC and BC + FA treatments, reflecting enhanced aromatization.^20^ The peaks at 1420 (N–H bending) and 1060 cm^−1^ (C–O stretching) were linked to polysaccharide and amide III of proteins, respectively.^9^ Notably, the BC + FA treatment altered the structural composition of key compost constituents (starch, protein and lipid), thereby promoting humification and maturation.

The Mantel test analysis further clarified the relationship between the dissolved index of the compost, NH_3_ vitalization, compost quality and GI (Fig. 7c). The results showed that the NH_3_ volatilization was negatively correlated with the C1, C4, HIX, DOC, HS and FA contents. It was positively associated with the BIX (*r* = 0.476, *P* = 0.006) and FI (*r* = 0.405, *P* = 0.018). The compost quality showed positive associations with all the dissolved indexes except C2, C4, BIX and HIX; however, none reached statistical significance. Specifically, GI exhibited the strongest correlations with C4 (*r* = 0.359, *P* < 0.019), HIX (*r* = 0.623, *P* = 0.002) and FA (*r* = 0.634, *P* = 0.004). Thus, C4 and HIX could emerge as key indicators in similar mixed composting systems.

## 4. Discussion

### 4.1. Effect of BC and FA as additives on ammonia volatilization in the compost

The NH_3_ volatilization serves as a critical indicator of composting efficiency and represents a major pathway for nitrogen loss during the composting process.^35,36^ It is generally positively correlated with the temperature, pH, and microbial activity.^15^

A peak of NH_3_ volatilization flux was observed during the thermophilic phase and declined gradually in the maturation phase (Fig. 2c). During the composting process, cumulative NH_3_ volatilization in the FA treatment was significantly reduced by 44.85% compared to the control (Fig. 2d). This reduction was probably attributed to the carboxyl and phenolic hydroxyl groups of FA, forming stable complexes with NH_4_^+^.^10,17^ Moreover, the addition of BC reduced the NH_3_ volatilization by 27.64% in this study. This could be attributed to its large specific surface area, which facilitated NH_3_ adsorption. The increased porosity mitigated anaerobic conditions, thereby creating a more favorable environment for nitrifying bacteria to metabolise NH_4_^+^.^37^ BC and FA reduced nitrogen loss effectively by adsorption or complexation.^38,39^

It was evidenced that the BC + FA treatment significantly reduced cumulative volatilization of NH_3_ by 48.11%. NH_3_ volatilization was also closely related to the content and type of nitrogen in the compost. Both BC and FA increased the total nitrogen content significantly in the compost (Fig. 4a), aligning with the previous findings.^40,41^ During the composting process, NH_4_^+^-N and NO_3_^−^-N exhibited dynamic fluctuations influenced by various processes. NH_4_^+^-N was identified as the primary contributor to NH_3_ volatilization, as evidenced by the SEM analysis (Fig. 3). Compared with CK, FA, BC and BC + FA treatments significantly reduced the NH_4_^+^-N contents by 17.57%, 22.16% and 20.12%, respectively.

In the early stage of composting, the mineralization of organic nitrogen resulted in an increase in NH_4_^+^-N, establishing it as the dominant form of inorganic nitrogen (Fig. 2a). Concurrently, the presence of anaerobic zones within the compost facilitated anaerobic bacteria in reducing NO_3_^−^-N to N_2_.^38,42^ By the seventh day, this reduction led to the lowest observed NO_3_^−^-N levels across all treatments (Fig. 2b). As the decomposition of organic nitrogen slowed and aerobic conditions improved, the activity of nitrifying bacteria increased. These bacteria oxidize NH_4_^+^-N to NO_3_^−^-N, leading to a decline in NH_4_^+^-N levels and a concurrent accumulation of NO_3_^−^-N (Fig. 2). This evidenced that BC treatment increased the NO_3_^−^-N content by 4.46% on day 28 compared to CK.

During the initial heating and thermophilic phases, EC acted as another significant factor promoting NH_3_ volatilization (Fig. 3). As composting progressed into the maturation phase, NH_4_^+^-N was gradually nitrified to NO_3_^−^-N, and the accumulation of NO_3_^−^-N became the primary driver of the increase in EC (Fig. 3d). Concurrently, high salinity might influence NH_3_ release through ion competition.^38^ The presence of other cations (*e.g.*, K^+^ and Ca^2+^) presumably competed with NH_4_^+^ for adsorption sites, leaving more NH_4_^+^ available in the solution phase.^20^ These mechanisms collectively explained why the BC + FA treatment effectively reduced NH_3_ volatilization (Fig. 2d). With the NH_3_ volatilization pronounced at the thermophilic phase, the BC + FA treatment maintained lower pH, EC, and NH_4_^+^-N levels in the compost, thereby mitigating the release of NH_3_ (Fig. 1 and 2).

The correlation between carbon metabolism and nitrogen decomposition has been documented, underscoring a synergistic relationship between nitrogen conversion and humification during kitchen waste composting.^43^ Accordingly, the stability of organic matter in the compost was crucial.^44^ As illustrated in Fig. 7c, NH_3_ volatilization was negatively correlated with the C1, C4, DOC, FA, and HS contents. Furthermore, the addition of HA was shown to reduce NH_3_ volatilization significantly by suppressing denitrification.^45^ In contrast, NH_3_ volatilization was positively correlated with the BIX (*P* < 0.01) and FI (*P* < 0.05), probably due to the enhanced microbial activity.^46^ Thus, the component and content of organic matter were key factors controlling NH_3_ volatilization. Consequently, BIX and FI could serve as reliable indicators for predicting maturity in the mixed manure compost.

### 4.2. Effect of FA and BC on the physiochemical parameters of composts

The rapid temperature increase observed on the second day of composting could probably be attributed to the proliferation of thermophilic bacteria and the swift decomposition of small molecules (Fig. 1a).^47^ The elevated temperatures recorded in BC and BC + FA treatments during the thermophilic and early maturation phases were probably due to the large specific surface area of BC. Previous studies have found which specific surface area provided favorable habitat for microorganisms and promoted organic matter degradation.^15^ In contrast, the addition of acidic substances such as FA might lower the compost pH and inhibit the microbial activity, resulting in a lower effective accumulated temperature observed in the BC + FA treatment than in the BC treatment alone (Fig. 1a).^48^

Consistent temperature fluctuations observed across all treatments prior to stabilization were primarily caused by periodic turning. This practice promoted uniform composting by redistributing organic matter and microbes.^47^ These findings aligned with previous studies,^1,2^ highlighting the critical role of aeration and mixing in achieving uniform compost maturation.

The increase in pH during the initial composting stage was primarily driven by organic matter decomposition and NH_3_ release *via* ammonification.^47^ BC's inherent alkaline further elevated the pH levels in the BC treatment after day 13. By the maturation phase, the compost in BC treatment exhibited significantly higher pH levels than that of CK, demonstrating its buffering capacity and role in sustaining an alkaline environment (Fig. 1b).^48^

EC reflected soluble salt content and was commonly used to evaluate the compost phytotoxicity.^7^ The EC of all treatments declined to its lowest point by day 4 (Fig. 1c), probably due to the rapid shift from weakly acidic to weakly alkaline conditions. This change promoted the precipitation of inorganic nutrients and metal ions. Subsequently, EC rose sharply from day 4 through the thermophilic phase, followed by a gradual increase during the maturation, potentially resulting from the slower degradation of organic matter.^47^ By the end of composting, BC showed significantly lower EC than both CK and FA (Fig. 1c). This reduction might be attributed to BC's ability to stabilize ions through pH elevation^49^ and adsorption.^1^ Together, these results indicated that BC could effectively reduce phytotoxicity and minimize the associated environmental risks.

The C/N ratio and GI are widely recognized as reliable indicators of compost maturity.^44^ By the end of composting process, the value of the C/N ratio across all treatments ranged from 17.14 to 20.61 (Fig. 4e), confirming that the composts had reached a mature state. The treatment of FA, BC and BC + FA reduced the C/N ratio by 16.85%, 12.18% and 2.76%, respectively, compared to CK. It was suggested that a GI value exceeding 50% indicated mature compost, while a GI above 80% was generally preferred to ensure lower phytotoxicity.^50^ In this study, the GI values of the CK and FA treatments were 66.77% and 64.68% (Fig. 4f), respectively, which remained below the 80% threshold considered safe for plant growth. In contrast, BC treatment exhibited a significantly higher GI (98.42%), confirming its suitability for plant use without phytotoxic risks. Previous studies have demonstrated that BC enhanced GI through multiple mechanisms, such as improving composition, stimulating microbial activity, reducing toxic substances, and increasing nutrient content.^11^ Conversely, the FA treatment showed no significant improvement in GI compared to CK. This might be attributed to the persistently high EC observed in both CK and FA at the end of the composting process (Fig. 1c). The higher EC increased salinity and the presence of harmful chemicals, thereby impairing water uptake and seed germination.

HSs served as the primary reservoir of organic carbon in soil and play a critical role in enhancing soil structure and nutrient retention.^51^ Due to its stable and highly aromatic carbon structure, BC exhibited limited conversion into HAs within a short composting period.^52^ Consequently, the HA content in the BC treatment was lower than that in the control (Fig. 5a). Within the HS fraction, HA exerted a more pronounced influence on long-term soil health.^53,54^ In contrast, FA was more effective in facilitating short-term plant nutrient uptake due to its higher bioavailability and susceptibility to microbial transformation.^27,53^

Generally, the mature compost is characterized by a higher ratio of HA/FA.^55^ In this study, FA treatment significantly increased the total HS content by 12.58% (Fig. 5a), whereas the BC treatment did not induce notable changes. An increase in HA content typically suggested a higher degree of humification and reflected enhanced compost maturity.^56^ In the present study, the FA content increased significantly by 8.08% in the treatment of FA compared to CK, whereas it decreased notably by 14.73% and 11.69% in the BC and BC + FA treatments, respectively. It was demonstrated that BC promoted the conversion of metals complexes initially associated with FA into those bound HA. This aligned with the previous observations of decreased FA and increased HA contents in the BC and BC + FA treatments (Fig. 5).^57^

Furthermore, the functional groups on BC contributed to the strong adsorption and complexation of HA,^58^ thereby enhancing the HA retention in composts. As a result, the HA/FA contents in both BC and BC + FA treatments significantly increased by 30.51% and 56.77%, compared to CK (Fig. 5). Moreover, the BC + FA treatment significantly increased the GI by 50%, and the Mantel test indicated a close correlation between the FA content and GI (Fig. 7). Therefore, these results suggested that BC addition promoted compost maturity and stability, ultimately improving the compost quality.

### 4.3. Key mechanism influencing composts under biochar or/and fulvic acid

DOM is produced during the decomposition of chicken manure and straw and served as a biostimulant that activates the microbial activity in composting.^59^ Regarding the DOM composition (Fig. 6), FA treatment increased the intensities of C1, C3, and C4 by 10.56%, 15.12%, and 30.21%, respectively, while the BC treatment decreased them by 17.89%, 15.51%, and 16.91%, respectively.^60^ The Mantel test result indicated a correlation between C4 and both NH_3_ emission and GI (Fig. 7). The changes in DOM during composting reflected ongoing degradation and transformation processes, which shaped the initial materials and composting conditions.^9,34,59^ Therefore, the addition of FA or/and BC altered the initial composting environment.

These DOM dynamics were associated with the microbial activity. The canonical correlation analysis (CCA; Fig. S1) showed that CK and BC + FA treatments were separated along CCA1, which was strongly correlated with the BIX (*r* = 0.837) and associated with NH_3_, GI, and compost quality. BC and FA treatments were further distinguished along CCA2, which was associated with HIX (*r* = −0.629). This result suggested that the importance of DOM-based indices in composting systems amended with BC and FA. In this study, the combined BC + FA treatment increased BIX (Fig. 6b), indicating that the DOM in composts was more influenced by biological activity than by external organic matter inputs.^46^ This implies that the increase in DOM primarily resulted from enhanced microbial degradation and transformation of organic matter.^59^

The FI index reflected the ratio of aromatic amino acids to non-aromatic compounds in DOM and provided insights into the origin and decomposition stage of HSs.^61^ The BC treatment significantly increased FI (Fig. 6b), probably due to the highly aromatic nature of BC, introducing additional aromatic compounds into the compost.^38^ This enhancement was further clarified by the Mantel test, which linked NH_3_ volatilization to BIX, which was itself an indicator of microbial activity.^20^

Moreover, the BC treatment slightly enhanced compost humification by increasing the HIX, whereas FA reduced it. The HIX reflected the humification degree of DOM and indicated the relative content and molecular complexity of HSs.^62^ Therefore, these findings made the HIX useful for monitoring and managing compost maturation.

## 5. Conclusion

The present study demonstrated that the addition of FA and BC to the process of aerobic composting effectively mitigated the ammonia emissions. It was observed that the co-utilization of FA and BC resulted in a significant reduction in ammonia volatilization. Meanwhile, the results indicated that both the efficiency of the composting process itself and the quality of the final product were enhanced. Moreover, with respect to the final products, FA increased the level of DOM, while BC promoted its humification. The combination of the additives resulted in elevated levels of nitrogen, organic matter and the germination index, which were found to be significantly greater than those observed with single additive treatments. Furthermore, the alternations in HA, BIX, HIX, and FI of the compost demonstrated the synergistic enhancement provided by the combination of BC and FA. It could thus be concluded that the co-utilization of BC and FA was more efficacious for the eco-utilization of livestock manure. It was evident that both additives were produced from bio-waste. Consequently, their combined role in composting could be regarded as a closed-loop strategy for the composting of waste biomass.

## Conflicts of interest

There are no conflicts to declare.

## Supplementary Material

RA-016-D5RA08201C-s001

## Data Availability

All data supporting the findings of this study are available within the article and its supplementary information (SI). Supplementary information is available. See DOI: https://doi.org/10.1039/d5ra08201c.
