# Supplementary material for: A closed-loop strategy for composting: using biochar and fulvic acid derived from manure or straw to mitigate ammonia emission and enhance humification
Source: RSC Adv. 2026 Feb 3;16(8):7149–62. doi: 10.1039/d5ra08201c (PMC12865790; doi:10.1039/d5ra08201c)

Supplementary Information (SI) for RSC Advances.
This journal is © The Royal Society of Chemistry 2026

Supplementary Materials

A Closed-Loop Strategy for Composting: Using Biochar and Fulvic Acid Derived to Mitigate Ammonia Emission and Enhance Humification

Chihao Yang [a, #], Jing Wu [c, #], Wenping Xie [a, *], Jingsong Yang [a], Yanfang Feng [b], Xiangping Wang [a], Xin Zhang [a], Xuan Yu [a], Rongjiang Yao [a]

[a] *State Key Laboratory of Soil and Sustainable Agriculture, Institute of Soil Science, Chinese Academy of Sciences, Nanjing 210008, China*

[b] *Key Laboratory of Agro-Environment in Downstream of Yangtze Plain, Institute of Agricultural Resources and Environment, Jiangsu Academy of Agricultural Sciences, Nanjing 210014, China*

[c] *College of Energy and Environment, Anhui University of Technology, Maanshan 243002, China*

[#] *These authors contributed equally to this work.*

**Corresponding author.*

Email address: *wpxie@issas.ac.cn*

Text S1 Extraction process of HSs, fulvic acid and humic acid

To extract HSs, 2.50 g of air-dried compost was mixed with 50 mL of an alkaline extractant (0.1M NaOH and 0.1M $Na_4P_2O_7 \cdot 10H_2O$ in a 1:1 volume ratio). The mixture was shaken at 25°C and 180 rpm for 16 hours. After shaking, the suspension was transferred to a centrifuge tube and centrifuged at 6,000 rpm for 10 minutes to separate the supernatant. This extraction process was repeated three times, with all supernatants combined and filtered to obtain the HSs extract. The pH of the extract was then adjusted to 1.0 by slowly adding 6M HCl solution, and the mixture was left undisturbed overnight. The following day, after centrifugation, the supernatant contained fulvic acid, while the precipitate contained humic acid. The precipitate was then washed with 0.01M HCl until the filtrate became colorless.

Fig.S1 Principal components analysis results for dissolved fraction parameters of compost with the NH_3 volatilization, GI and compost quality.

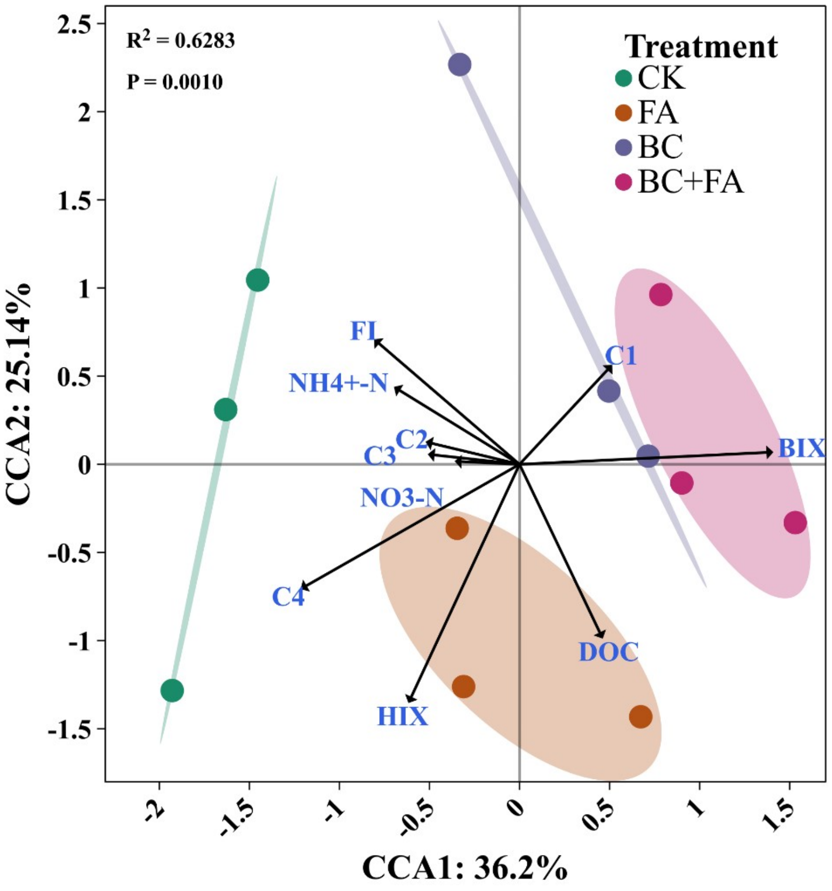

Supplement: RA-016-D5RA08201C-s001 [file RA-016-D5RA08201C-s001.pdf]
